# Unveiling the Influence of Linkers on Conformations of Oligomeric Acceptors for High‐Performance Polymer Solar Cells

**DOI:** 10.1002/advs.202406772

**Published:** 2024-08-29

**Authors:** Jingnan Wu, Fengbo Sun, Xunchang Wang, Qiaonan Chen, Leandro R. Franco, Xufan Zheng, C. Moyses Araujo, Renqiang Yang, Donghong Yu, Ergang Wang

**Affiliations:** ^1^ Department of Chemistry and Chemical Engineering Chalmers University of Technology Göteborg SE‐412 96 Sweden; ^2^ Department of Chemistry and Bioscience Aalborg University Aalborg DK‐9220 Denmark; ^3^ Key Laboratory of Optoelectronic Chemical Materials and Devices (Ministry of Education) School of Optoelectronic Materials & Technology Jianghan University Wuhan 430056 China; ^4^ Department of Engineering and Physics Karlstad University Karlstad 65188 Sweden; ^5^ Materials Theory Division Department of Physics and Astronomy Uppsala University Uppsala 75120 Sweden; ^6^ Sino‐Danish Center for Education and Research Aarhus DK‐8000 Denmark

**Keywords:** molecular conformation, oligomeric acceptors, solar cells

## Abstract

Conformational isomerism of organic photovoltaic materials has a profound impact on their molecular packing and therefore performance of polymer solar cells (PSCs). However, the conformations of oligomeric acceptors (OAs) are mostly predicted by simulations rather than experimental determinations. Herein, the stereochemical *S*‐shaped structure of two dimeric‐type acceptor molecules, V‐DYIC and V‐DYIC‐4F, is first confirmed with different end groups (IC for V‐DYIC and IC‐2F for V‐DYIC‐4F), incorporating vinylene linkage for connecting the distinct state‐of‐the‐art small molecule acceptor *Y*‐segments. Through the synthetic control of fluorination sites adjacent to the vinyl‐linker, *S*‐shaped the conformation by NMR experiments is validated. Compared to the *O*‐shaped dimer, *S*‐shaped conformation results in enhanced lamellar order and reduced nonradiative recombination losses. The optimal acceptor, V‐DYIC‐4F, achieved a champion efficiency of 18.10% with the lowest energy loss of 0.556 eV in its devices paired with PM6 due to their efficient carrier transport, and suppressed recombination compared to other devices, being attributed to the synergistic effect of conformation and end group fluorination. The insights gained in this work contribute valuable knowledge of both synthetic control and structural determination of OAs, providing strategic design guidelines for the future development of dimeric acceptors toward high‐efficiency PSCs.

## Graphical abstract

Two dimeric‐type acceptors, namely V‐DYIC and V‐DYIC‐4F with vinylene linkage, are synthesized and reported to have a rigid and coplanar *S*‐shaped conformation via NMR and DFT calculations. The *S*‐shaped V‐DYIC‐4F‐based solar cell demonstrated an excellent PCE of 18.10% with lower nonradiative recombination loss when compared to DIBP3F‐S with an *O*‐shaped conformation.

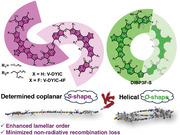

## 1 Introduction

Recently, oligomeric acceptors (OAs), composed of a certain number of Y6‐type structural repeating units, have emerged as a novel class of acceptor molecules, garnering significant attention in the field of polymer solar cells (PSCs) due to their precisely defined structures and elimination of batch‐to‐batch variations compare to their polymer acceptors (PAs) counterparts.^[^ ^1^, ^2^ ^]^ Moreover, they exhibit superior device‐ performance and ‐stability,^[^ ^3^, ^4^, ^5^ ^]^ mitigating morphological degradation seen in small‐molecule acceptors (SMAs).^[^ ^6^, ^7^, ^8^ ^]^ Leveraging the advantages of SMAs and PAs, OAs exhibit exceptional optoelectronic properties, featuring an absorption in visible and near‐infrared ranges with a higher absorption coefficient and enhanced electron mobility with moderate molecular packing. However, compared with extensive studies on either SMAs or PAs that have markedly improved their PCEs,^[^ ^9^, ^10^, ^11^, ^12^, ^13^, ^14^, ^15^, ^16^, ^17^, ^18^ ^]^ the development of OAs significantly lags behind with few reports with PCEs of >15% in their PSCs achieved. Consequently, there is an urgent need for more concerted efforts in capitalizing OAs’ advantages for enhanced photovoltaic performance of PSCs.

Oligomerization of Y6 derivatives through direct coupling of the SMA segments was pioneered by He et al. in 2022.^[^ ^4^ ^]^ Their study demonstrated that such OA‐based devices exhibited enhanced PV performance and improved device stability retaining 80% of their initial PCE under illumination for 1020 h superior to those utilizing either SMA‐ or PA‐based materials. However, the oligomers suffer distortion in the backbone driven by the conformational isomerism through the rotation of sp^2^ hybridized C‐C σ‐bonds between the adjacent Y6‐blocks, rendering them unsuitable for establishing ordered intermolecular packing and effective charge transport pathways.^[^ ^1^, ^19^ ^]^ To overcome this challenge, researchers utilized a linker, particularly a rigid conjugated spacer, during oligomerization to obtain enlarged planar conjugated structures.^[^ ^1^ ^]^ For instance, the incorporation of a thiophene π‐linker decreased the torsional angle from 35° (indirect linking) to less than 20°.^[^ ^5^, ^20^ ^]^ Furthermore, modifying the π‐linkage with double fluorinated thiophene resulted in an even smaller twisting angle of 12°, facilitated by S···F noncovalent interactions.^[^ ^20^ ^]^ Among the different linkers investigated, the fluorinated one exhibited a planar geometry, red‐shifted absorption, and a more compact π–π stacking arrangement.^[^ ^20^, ^21^ ^]^

To date, more attention has been drawn to the planarity of molecules indicated by density functional theory (DFT) simulations, rather than the conformational complexities inherent in OAs.^[^ ^4^, ^5^, ^21^, ^22^, ^23^, ^24^ ^]^ The introduction of rotating σ‐bonds invariably engenders the formation of conformers. However, the impact of these different conformations on intermolecular packing behavior, morphological characteristics, charge transport properties, and overall device performance has been rarely investigated. Li et al. explored the effects of regio‐isomerization on photophysical properties, molecular conformation, and PV performance of two dimeric acceptors, EV‐i in *U*‐shape and EV‐o in *S*‐shape conformation, respectively.^[^ ^24^ ^]^ That *U*‐shaped EV‐i exhibited a twisted molecular conformation but enhanced conjugation, while *S*‐shaped EV‐o showed a better planar molecular structure but weakened conjugation. PSCs based on PM6:EV‐i demonstrated a remarkably higher PCE of 18.27%, compared to PM6:EV‐o (2.50%). It is important to note that these *S‐* and *U*‐shaped conformations were derived through theoretical calculations level of comparing potential energy, thus not providing concrete proof of their configurations. To address this limitation, our group tailor‐designed two dimers, and performed comprehensive 1D‐ and 2D‐NMR experiments to accurately validate their *O*‐shaped conformation, deviating from the conventional *S*‐ or *U*‐shaped structures.^[^ ^19^ ^]^ This highlights the significance of investigating the impact of linkers on real molecular conformation of OAs, an intriguing and relatively understudied aspect within the realm of PSCs.

**Figure 1:**
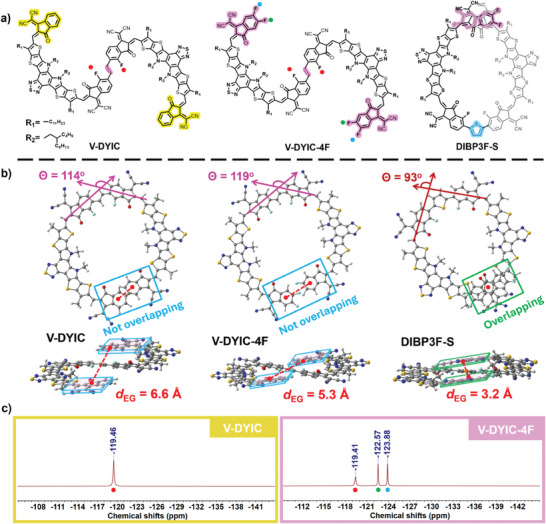
a) Chemical structures of V‐DYIC, V‐DYIC‐4F, and DIBP3F‐S. b) The top‐ and side‐view of *O*‐shaped V‐DYIC, V‐DYIC‐4F, and DIBP3F‐S. c) ^19^F NMR spectrum (600 MHz) of *S*‐shaped conformations of V‐DYIC and V‐DYIC‐4F in the C_2_D_2_Cl_4_ solution.

Herein, diverging from the commonly assumed *S*‐shaped conformations in literature,^[^ ^5^, ^21^, ^22^, ^23^, ^24^, ^25^ ^]^ we present the first evidence of the existence of conformation “*S*” by synthesizing two dimeric‐type acceptor materials, denoted as V‐DYIC and V‐DYIC‐4F. These materials incorporate vinylene linkage units, serving to bridge distinct *Y*‐segments (BPIC‐F and BPIC‐3F as shown in Scheme S1, Supporting Information (SI) respectively). Importantly, the strategic position‐selective fluorination of IC close to the linker provides a selective isomeric structure for the dimers, facilitating the determination of their conformation through ^19^F NMR and DFT calculations. To elucidate the consequential influence of molecular conformation, the reported *O*‐shaped DIBP3F‐S (**Figure** **1**a) with thiophene as a linkage was selected for comparison. The *S*‐shaped conformation observed in the vinylene‐linked acceptors demonstrates a more coplanar and rigid structure compared to the helical structure of the *O*‐shaped counterpart, with negligible torsional angles less than 2° between the linker and the adjacent *Y* moiety. As anticipated, V‐DYIC‐4F, featuring IC‐2F as the ending group (EG), exhibits a redshifted absorption, a downshift energy level, stronger π‐π stacking, and a more ordered molecular arrangement than the IC EG‐based one. This translates to increased short‐circuit current (*J*_sc_) and fill factor (FF), though with a decreased open‐circuit voltage (*V*_oc_). Notably, despite the *S*‐shaped conformation of V‐DYIC‐4F, which features a relatively low level lowest unoccupied molecular orbital (LUMO), it demonstrates a comparable *V*_oc_ of 0.883 V to that of the *O*‐shaped DIBP3F‐S (0.881 V). Further investigation reveals that an *S*‐shaped conformation contributes to enhanced lamellar orderliness, while an EG (di)fluorination strategy improves the strength of π–π stacking. V‐DYIC‐4F amalgamates both benefits from the two, exhibiting robust crystallinity in both in‐plane (IP) and out‐of‐plane (OOP) directions, which is confirmed by means of grazing incidence wide‐angle X‐ray scattering (GIWAXS) study. This leads to efficient carrier transport and suppresses recombination in their corresponding PSC devices. Additionally, energy loss (*E*_loss_) analysis indicates that dimers featuring the difluoride atoms of EG effectively suppress energy disorder, and the *S*‐shaped conformation minimizes nonradiative recombination loss. Consequently, when blended with the popular polymer donor PM6, the V‐DYIC‐4F‐based device demonstrates the best performance, achieving a champion PCE of 18.10% and the smallest *E*_loss_ of 0.556 eV among the three OAs‐based devices (15.78% and 0.558 eV for V‐DYIC and 16.06% and 0.573 eV for DIBP3F‐S).

## 2 Results and Discussion

The synthetic routes and detailed procedures for synthesizing V‐DYIC and V‐DYIC‐4F are presented in Scheme S1 in SI, and the Materials and Methods section in SI. A starting material of Y‐BO‐CHO was chosen for reacting with native‐ and bromo‐fluoro‐IC (IC‐FBr) into the key intermediate BTIC‐FBr through Knoevenagel condensation reaction with IC and IC‐FBr as asymmetric EGs. Subsequently, the target molecules of V‐DYIC and V‐DYIC‐4F were obtained through typical Stille coupling reactions based on the two mono‐brominated BTIC‐FBr and BTIC‐3FBr, respectively, along with a formation of *trans*‐vinylene linkers. All chemical structures of intermediates and target products were confirmed using ^1^H‐, ^13^C‐, ^19^F‐NMR, and MALDI‐TOF techniques. To gain insights into the molecular geometries of the two dimeric acceptors, DFT calculations were conducted at the B3LYP‐GD3BJ level. Due to the presence of a rotatable sp^2^ hybridized C─C σ‐bond between the linker and the adjacent IC moiety in BTIC‐3F (the *Y* block), three prominent isomeric conformations emerge, namely the *S*‐, *O*‐, and *γ*‐shape, as visually depicted in Figure S1 (SI). Among these, the *O*‐ and *γ*‐shapes adopt a helical structure in a side‐view, particularly the γ‐shape, which exhibits substantial dihedral angles exceeding 30° between the linker and *Y* block. In contrast, the *S*‐shape displays a predominantly co‐planar and rigid conformation, characterized by negligibly small dihedral angles of <2°, facilitating the formation of strong intermolecular stacking interactions.

Referencing the energetic analysis, as seen in Table S1 (SI), it was revealed that any conformation could potentially be accessed. Nevertheless, both OAs of V‐DYIC and V‐DYIC‐4F exhibited a tendency to eschew the *γ*‐shape conformation due to its significantly higher electronic energy. It is crucial to note that two dimers are synthesized through *trans*‐substituted ethylene, a factor more conducive to the formation of *trans*‐isomer,^[^ ^26^ ^]^ thereby excluding the possibility of cis‐ *γ*‐shaped conformations. Next, the theoretical chemical shifts (*δ*s)^[^ ^27^ ^]^ of the aromatic protons (labeled in Figure S2, SI) were compared with the experimental ones obtained in chloroform solution, as illustrated in Tables S2, S3 (SI). The *δ*s of the two dimers in the *S/O*‐shape conformation align well with the experimental trend, suggesting the coexistence of *O* and *S* conformations, respectively. Then, we turned to ^1^H‐^1^H Nuclear Overhauser Effect Spectroscopy spectra (Figures S3, S4, SI) to further prove the conformations of such products. Unfortunately, all conformations featuring the exchange cross‐peaks (labeled as A, B, and C) within the inter‐proton of H_a_‐H_b_, H_a_‐H_c_, and H_b_‐H_c_ (with the inter‐proton distances of less than 5 Å^[^ ^28^ ^]^ obtained), were not distinguishable using 2D NMR. In the end, the strategic introduction of the fluorine next to the linker allows us to distinguish the *S*‐ and *O*‐shaped conformers from the ^19^F NMR spectra. The *O*‐shaped conformer would expose the two F atoms next to the linker in different chemical environments and therefore two peaks can be expected. However, as shown in Figure 1c, only one peak can be identified, which indicates only an *S*‐shaped conformer exists in both V‐DYIC and V‐DYIC‐4F. Consequently, based on these meticulous investigations from both experiments and DFT simulations, we can assert that both V‐DYIC and V‐DYIC‐4F adopt the stable molecular conformation of the *S*‐shape.

Nonetheless, in our previous report on the conformation of the dimer of DIBP3F‐S (distinguishing the linker unit of thiophene instead of vinylene), we did conclude an emergence of the *O*‐shaped conformation as a result of robust intramolecular interactions between the two terminal IC moieties.^[^ ^19^ ^]^ While the current dimers of V‐DYIC and V‐DYIC‐4F adopt the *S*‐ rather than the *O*‐shaped conformation as an intriguing phenomenon, which could be attributed to the absence of a “conformational lock” arising from the close proximity of the EGs in the case of the *O*‐shaped conformation. For OAs in *O*‐shaped conformation, our current investigation reveals that replacing the thiophene linkage with a simpler (lesser conjugation region, i.e., lower electron density) vinylene unit could increase the distance between the EGs for V‐DYIC and V‐DYIC‐4F as compared to DIBP3F‐S. This is reasonably explained by the larger angle between the two connected IC moieties: 114°–119° for vinylene‐linked OAs and 93° for DIBP3F‐S (refer to the θ in Figure 1b). Therefore, the large distance of the two intramolecular EGs (*d*_EG_) in an *O*‐shaped conformer for V‐DYIC and V‐DYIC‐4F as shown in Figure 1b, results in the absence of conformational locking force. As for the formation of the *S*‐shape, it can be also explained by the synthesis process during the Stille coupling reactions between bis‐stannyl ethylene and mono‐brominated *Y* moieties, where the *S*‐shape is promoted by the formation of strong intramolecular F⋯H interactions between the F atom next to bromine from *Y* segment and the H atom from ethylene. To understand the differences in *S*‐ and *O*‐shaped dimers, we also included *O*‐shaped DIBP3F‐S as a comparative model for further studies. Such differences in the oligomer conformations may impact their optoelectronic properties, charge transport, recombination, morphology, and the performance of the PSCs, which will be discussed in more detail below.

**Figure 2:**
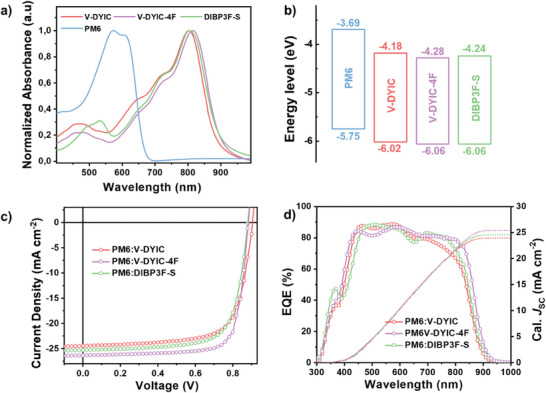
a) Normalized UV–vis absorption spectra in pristine films and b) energy levels of PM6, V‐DYIC, V‐DYIC‐4F, and DIBP3F‐S. c) J–V characteristics and d) EQE curves and integrated *J*_sc_ values for optimized devices based on PM6:V‐DYIC, PM6:V‐DYIC‐4F, and PM6:DIBP3F‐S.

The optical absorption spectra of the OAs, both as thin films and in chloroform solution, are presented in **Figure** **2**a and Figure S5 (Supporting Information), respectively. All acceptors present large, red‐shifted spectra from solutions to the solid states, which is attributed to their excellent molecular planarity and the intermolecular interactions induced by aggregation. Additionally, they display notable absorption extinction coefficient values of ≈2 × 10^5^ cm^−1^ (Table S4 and Figure S6, Supporting Information) at ≈810 nm in film. Notably, the film absorption maximum and onset of V‐DYIC‐4F (at 817 and 904 nm, respectively) are red‐shifted in contrast to those of DIBP3F‐S films (804/899 nm). This red shift is more pronounced than the previously reported 1 nm shift observed in dimer molecules (DYT and DYV),^[^ ^22^ ^]^ where the linker is changed from thiophene to vinylene without the presence of adjacent F atoms. The enhanced red shift observed in V‐DYIC‐4F can be attributed to the improved planarity of the *S*‐shaped conformation, indicating a stronger tendency for molecular stacking. This is supported by the closer π–π stacking observed from the GIWAXS results (Table S10, Supporting Information). Subsequently, we proceeded to evaluate the energy levels of the three OAs through cyclic voltammetry measurements (Figure 2b; Figure S7, Supporting Information). The estimated highest occupied molecular orbital (HOMO)/LUMO levels for V‐DYIC‐4F are determined to be −4.28/−6.06 eV, which are lower than those of V‐DYIC with nonfluorinated EG. This observation aligns with the findings from DFT calculations (Figure S8, Supporting Information). Moreover, *S*‐shaped V‐DYIC‐4F also exhibits slightly shifted downward energy levels when compared to the *O*‐shaped DIBP3F‐S with thiophene linker. The lower‐lying LUMO may potentially have a detrimental impact on the *V*_oc_ in PSC devices.

Then, the photovoltaic performance of three devices within a conventional architecture featuring ITO/PEDOT:PSS/PM6:OA/PFN‐Br/Ag was investigated. Detailed device processing information refers to Figure S9 and Tables S5, S6 (Supporting Information). The representative current–voltage (*J–V*) curves and the collected performance data are presented in Figure 2c and **Table** **1**, respectively. Notably, the device based on the optimized PM6:V‐DYIC‐4F blend exhibits the best PCE of 18.10%, with a *V*_oc_ of 0.883 V, a high *J*_sc_ of 26.35 mA cm^−2^, and a commendable FF of 77.8%. In contrast, the PM6: V‐DYIC‐based device, while showing an enhanced *V*_oc_ of 0.905 V, suffers from a limited light absorption range attributed to V‐DYIC's spectral response (Figure 2d), resulting in a lower *J*_sc_ of 24.46 mA cm^−2^ and a consequently reduced PCE of 15.78%. Interestingly, despite the slightly downshifted LUMO level of V‐DYIC‐4F, this does not translate into a lower *V*_oc_ (0.883 V) in the device compared to PM6:DIBP3F‐S. As displayed in Figure 2d, the device based on V‐DYIC‐4F presents a wider and more red‐shifted external quantum efficiency (EQE) response in the range of 300–950 nm, with a particularly higher response within the 600–950 nm wavelength range, corresponding to the highest integrated *J*_sc_ of 25.41 mA cm^−2^, as compared to V‐DYIC (23.97 mA cm^−2^) and DIBP3F‐S (24.57 mA cm^−2^). It is worth noting that the integrated *J*_sc_ values obtained from the EQE curves are consistent with those extracted from the *J–V* curves. Furthermore, the stability of the three PSCs was also evaluated. As depicted in Figure S10 (Supporting Information), following aging at 65 °C for 18 days, all three devices retained over 80% of their initial PCE. Extending the aging period to 28 days revealed that the systems based on V‐DYIC‐4F and DIBP3F‐S maintained efficiency levels above 75%, while the devices with V‐DYIC exhibited a notable decline to 62%, referring to their initial values. This interestingly indicates that the effect of molecular conformation on morphology stability is less pronounced compared to the impact of EG difluorination during the aging process, which is worthy of being further investigated in future work.

**Table 1:** Photovoltaic performance of the PSCs based on PM6: acceptor (1:1.2, w/w) under the illumination of AM 1.5 G, 100 mW cm^−2^.

D:A	*V*_oc_ [V]	*J*_sc_ [mA cm^−2^]	Cal. *J*_sc_ ^a^ ^)^ [mA cm^−2^]	FF [%]	PCE_max_^/^PCE_avg_ ^b^ ^)^ [%]
PM6:V‐DYIC	0.905	24.46	23.97	71.2	15.78 (15.67 ± 0.11)
PM6:V‐DYIC‐4F	0.883	26.35	25.41	77.8	18.10 (17.95 ± 0.13)
PM6:DIBP3F‐S	0.881	25.31	24.57	72.1	16.06 (15.93 ± 0.10)

^a)^ The integral *J*_sc_ in brackets were calculated from the EQE curves

^b)^ The average PCE values were obtained from 15 devices with their deviations shown in brackets.

**Figure 3:**
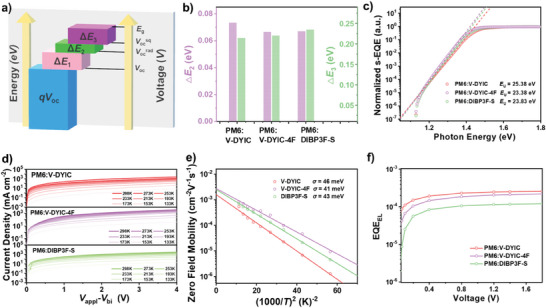
a) Diagram describing voltage and energy loss. b) Δ*Ε*_2_ and Δ*Ε*_3_ energy loss, c) s‐EQE at absorption onset, d) temperature‐dependent space‐charge‐limited current (SCLC) curves, e) electron mobility of corresponding devices as a function of 1/*T* ^2^ using SCLC estimated data, and f) electroluminescence EQE of optimized devices based on PM6:V‐DYIC, PM6:V‐DYIC‐4F, and PM6:DIBP3F‐S.

Considering the disparity in *V*_oc_ observed among the three devices, *E*_loss_ analysis was conducted, and the corresponding values are summarized in Table S7 (Supporting Information). Following previous studies,^[^ ^29^, ^30^, ^31^ ^]^ the *E*_loss_ can be partitioned into three components (Δ*Ε*_1_, Δ*Ε*_2_, and Δ*Ε*_3_ in **Figure** **3**a). Generally, Δ*Ε*_1_ (*E*_g_ ‐*qV*_oc_^SQ^, where *V*_oc_^SQ^ is the maximum voltage calculated by the Shockley–Queisser limit) is an unavoidable loss and typically falls within the range of 0.25–0.30 eV. It was noted that the voltage variations primarily stem from fluctuations in Δ*Ε*_2_ and Δ*Ε*_3_ as illustrated in Figure 3b. The Δ*Ε*_2_ values, associated with radiative recombination originating from absorption below the band gap, are determined to be 0.074, 0.067, and 0.067 eV for PM6 blending with V‐DYIC, V‐DYIC‐4F, and DIBP3F‐S, respectively. It is noteworthy that the lower Δ*Ε*_2_ for the fluorinated EG‐based devices related to the more ordered molecular packing is comparable to that of PSC based on the nonfluorinated counterpart. To explain the different Δ*Ε*_2_ loss, the Urbach energy (*E*_U_),^[^ ^32^ ^]^ serving as an indicator of energy disorder, was first measured using highly sensitive external quantum efficiency (s‐EQE) measurements (Figure 3c).^[^ ^33^ ^]^ It is evident that the devices based on V‐DYIC‐4F and DIBP3F‐S exhibit the lowest *E*_U_ value at 23.38 and 23.83 meV, respectively, while PM6:V‐DYIC displays a larger one at 25.58 meV. The smaller *E*_U_ values imply a reduction in energetic disorder by incorporating F atoms on their EGs, which can be attributed to the higher crystallinity and more ordered packing of materials within the blend films.

To gain deeper insights into the disorder within the blend film, the electron energetic disorder (*σ*) was also determined through temperature‐dependent electron transport measurements (Figure 3d) using the Gaussian disorder model (GDM),^[^ ^34^ ^]^ as expressed in the following formula: *µ*_0_ = *µ*∞ exp [‐(2*σ*/3*kT*)^2^], where *k* represents the Boltzmann constant, *T* denotes the temperature in Kelvin, *µ∞* signifies the mobility at infinite temperature. Figure 3e illustrates the plots of electron zero‐field mobilities of the two devices as a function of 1/*T* ^2^ along with the corresponding fitted linear curves. The decreasing σ values in the order of V‐DYIC (*σ* = 46 meV), DIBP3F‐S (*σ* = 43 meV), and V‐DYIC‐4F (*σ* = 41 meV) indicate a lower density of trapped states within those in the blends of PM6/V‐DYIC‐4F and PM6/DIBP3F‐S. This, in turn, suggests that holes have a reduced likelihood of becoming trapped at tailed states and subsequently recombining with electrons. Consequently, PM6:V‐DYIC‐4F and PM6:DIBP3F‐S blend exhibit significantly fewer traps and lower energy disorder. Based on these results, it is evident that acceptors featuring peripheral fluorine exhibit lower energy disorder, which corresponds to a reduced Δ*Ε*_2_.

The Δ*Ε*_3_, representing nonradiative *E*_loss_, can be calculated using the equation: Δ*Ε*_3_ = ‐*kT*ln(EQE_EL_). As shown in Figure 3f, the electroluminescence EQE (EQE_EL_) measurements indicate that the emission efficiency of the PSCs based on V‐DYIC and V‐DYIC‐4F, highlighting *S*‐shape skeleton, are 2.37 × 10^−2^% and 1.88 × 10^−2^% (measured with injection current equal to *V*_oc_), respectively, which is significantly higher than that observed in the *O*‐shaped DIBP3F‐S‐based counterpart (1.07 × 10^−2^%). This suggests that dimeric acceptors featuring the *S*‐shape conformation exhibit higher emission efficiency along with lowered Δ*Ε*_3_. Unquestionably, the reductions in both Δ*Ε*_2_ and Δ*Ε*_3_ contribute to the overall reduction in *E*_loss_, amounting to 0.556 eV for V‐DYIC‐4F‐based PSCs. These results underscore that the incorporation of the F atoms on IC moiety and the adoption of the *S*‐shape conformation for OAs effectively mitigate both Δ*Ε*_2_ and Δ*Ε*_3_. This, in turn, leads to suppressed energy disorder and minimizes nonradiative recombination losses.

**Figure 4:**
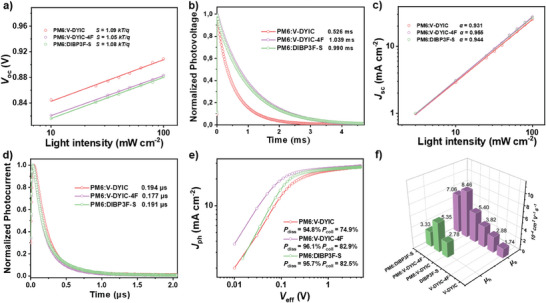
a) Light intensity dependency on *V*_oc_, b) Normalized TPV data, c) light intensity dependency on *J*_sc_, d) Normalized TPC data, e) characteristics of the photocurrent density versus effective voltage (*J*_ph_‐*V*_eff_) for the optimized PSCs. f) Hole and electron mobilities of the devices obtained from SCLC measurements for, solar cell devices from PM6:V‐DYIC, PM6:V‐DYIC‐4F, and PM6: DIBP3F‐S blends, respectively.

In addition to maintaining relatively high *V*_oc_ and low voltage/energy loss, EG fluorination‐based OA with *S*‐shape conformation also exhibits excellent *J*_sc_ and FF. Further investigations into carrier transport and recombination were undertaken to elucidate the mechanism contributing to the enhanced photovoltaic performance observed in the V‐DYIC‐4F‐based device. Recombination properties within the three devices were examined by analyzing photovoltaic parameters under varying light intensities (*P*_light_). By fitting the *V*_oc_s with light power on a semilogarithmic scale (*V*_oc_ ∝ n*kT/q* ln*P*_light_, **Figure** **4**a), the slopes (*S*) were close to *kT/q* for the three cells, implying effectively suppressed trap‐mediated charge recombination in their devices.^[^ ^35^ ^]^ In Figure 4b, photovoltage decay kinetics are primarily governed by charge recombination using transient photovoltage (TPV) measurement, and the decay time reflects the lifetimes of the charges.^[^ ^36^ ^]^ Devices utilizing V‐DYIC‐4F or DIBP3F‐S exhibit longer lifetimes (1.039 and 0.990 ms, respectively) compared to the V‐DYIC‐based one (0.526 ms). This observation is further supported by irradiation‐dependent *J*_sc_ measurements, as shown in Figure 4c, indicating a power‐law dependency on the power (*J*_sc_ ∝ *P*_light_^𝛼^) approaching unity.^[^ ^37^ ^]^ This implies that bimolecular recombination is indeed the dominant process. Therefore, the longer charge lifetime in devices based on OAs with EG fluorination can be attributed to the suppression of bimolecular recombination.

In Figure 4d, photocurrent decay kinetics under short‐circuit conditions are probed through transient photocurrent (TPC).^[^ ^36^ ^]^ It is evident that the V‐DYIC‐4F‐based cell exhibits faster decay, indicating a swifter carrier sweepout time compared to V‐DYIC and DIBP3F‐S. The charge extraction of the V‐DYIC‐4F device (0.177 µs) is notably shorter than that of the V‐DYIC (0.194 µs) and DIBP3F‐S (0.191 µs), respectively, indicating more efficient charge extraction. In summary, the TPV and TPC results collectively suggest that the PM6:V‐DYIC‐4F BHJ exhibits reduced recombination and more efficient charge extraction. In addition, the exciton dissociation probabilities (*P*_diss_, under *J*_sc_ conditions) and charge collection efficiencies (*P*_coll_ at the maximum power point) were calculated as 94.8%/74.9%, 96.1%/82.9% and 95.7%/82.5% for the PM6:V‐DYIC, PM6:V‐DYIC‐4F and PM6: DIBP3F‐S blends, respectively (Figure 4e). The higher *P*_diss_ and *P*_coll_ values observed in PM6:V‐DYIC‐4F‐based PSCs signify superior exciton dissociation and enhanced charge collection, which, in turn, can account for the higher *J*_sc_ and FF values in the corresponding devices.^[^ ^38^, ^39^ ^]^

Charge transport characteristics in both neat and blend films were evaluated by space‐charge‐limited current (SCLC) measurements^[^ ^40^ ^]^ (Figure 4f; Table S8, Supporting Information). All neat/blend films based on OAs with difluorinated IC present higher electron mobilities (*µ*_e_) (2.88/8.46 × 10^−4^ and 3.82/7.06 × 10^−4^ cm^2^ V^−1^ s^−1^ for V‐DYIC‐4F and DIBP3F‐S, respectively) compared to the nonfluorinated one (1.74/5.40 × 10^−4^ cm^2^ V^−1^ s^−1^ for V‐DYIC). This outcome underscores that the acceptors containing IC‐2F moieties demonstrate enhanced electron conductivity when compared to their nonfluorinated counterparts, a result that aligns with expectations. Moreover, PM6:V‐DYIC‐4F films exhibit more balanced electron and hole mobilities, as reflected in a *µ*_e_/*µ*_h_ ratio of 1.58, whereas those of 1.94 and 2.12 for PM6:V‐DYIC and PM6:DIBP3F‐S blended films display substantial imbalances. That unbalanced charge transport can reasonably account for the lower FF observed in their devices. These results highlight the efficacy of fluorinated EG modification and the advantages of the *S*‐shape conformation in suppressing trap‐mediated recombination, promoting efficient electron transport and high exciton dissociation efficiency, and ultimately maximizing the PSC performance.

By means of contact angle measurements (Figure S11, Supporting Information), surface free energy (*γ*)^[^ ^41^ ^]^ values were determined as 40.47, 45.18, 41.22, and 39.16 mN m^−1^ for neat films of PM6, V‐DYIC, V‐DYIC‐4F, and DIBP3F‐S, (Table S9, Supporting Information), respectively. Notably, the introduction of peripheral F atoms to dimeric acceptors contributes to a reduction in *γ*. This effect is reflected in the lower interaction parameters (*χ*) calculated between PM6 and acceptors (V‐DYIC and V‐DYIC‐4F) using the Flory–Huggins method,^[^ ^42^, ^43^ ^]^ signifying enhanced miscibility between the donor and acceptors. Lower χ values (according to the equation *χ* ∝ (*γ*_D_^−2^‐*γ*_A_^−2^)^2^), estimated as 0.1296, 0.0107, and 0.0034 for PM6‐based blends of V‐DYIC, V‐DYIC‐4F, and DIBP3F‐S, respectively, suggest reduced potential for excessive phase separation and favorable conditions for exciton dissociation, particularly in the aforementioned order. However, V‐DYIC‐4F exhibits a relatively weaker compatibility with PM6 as reflected by its slightly bigger *χ* value compared to DIBP3F‐S. This propensity promotes the formation of interpenetrating networks with the more appropriate (enhanced) size of fiber‐like domains as shown in the atomic force microscopy (AFM) phase images (Figure S12, Supporting Information), which is consistent with the highest *P*_diss_ and *P*_coll_ of the PM6:V‐DYIC‐4F blend as discussed above from the TPC and TPV analysis.

**Figure 5:**
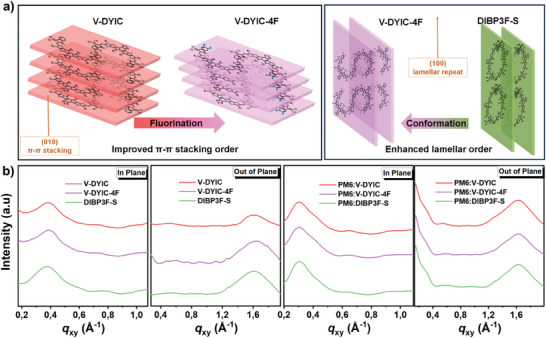
a) The schematic π–π stacking of V‐DYIC and V‐DYIC‐4F (left), and the schematic lamellar packing of V‐DYIC‐4F and DIBP3F‐S (right). b) 1D line cuts of the films based on neat V‐DYIC, V‐DYIC‐4F, and DIBP3F‐S, and their blends with PM6.

To reveal the nuanced variations in the morphology of blend films prompted by both EG modification and conformational effects, GIWAXS characterization was conducted to scrutinize the crystallinity and orientation of films (Figures S13, S14, and Table S10, Supporting Information).^[^ ^44^, ^45^ ^]^ The neat and blended films exhibit a discernible lamellar stacking peak in the IP direction and a dominant π–π stacking peak in the OOP direction at the range of 0.31–0.38 and 1.61–1.63 Å^−1^, respectively. This implies a face‐on orientation conducive to vertical charge transport. Regarding neat OAs, V‐DYIC, and V‐DYIC‐4F manifest slightly denser *d*‐spacing corresponding to the (100) plane, coupled with markedly enhanced crystal coherence length (*CCL*) values of 56.54 and 61.65 Å, respectively, compared to that of 42.77 Å for DIBP3F‐S in the IP direction. This suggests a more ordered lamellar packing for the molecules with *S*‐shaped conformation. (**Figure** **5**a). In the OOP direction, V‐DYIC exhibits subdued peaks at *q* = 1.61 Å⁻¹ (*d* = 3.89 Å) with a *CCL* value of 20.33 Å, which is a bit surpassed by the V‐DYIC‐4F at *q* = 1.63 Å⁻¹ (*d *= 3.84 Å) and an enhanced *CCL* of 21.21 Å. This divergence may be attributed to the F atoms facilitating the formation of multiple noncovalent interactions, promoting deeper intermolecular overlap and tighter layer‐layer contact. Upon blending with PM6, the three blends exhibit identical lamellar stacking peaks at 0.31 Å⁻¹ (ascribed to the donor, see Figure S15 and Table S8, Supporting Information) in the IP direction, indicating minimal influence from different OAs on PM6 molecular packing. However, a noteworthy increase in *CCL* is observed from the *S*‐shaped V‐DYIC and V‐DYIC‐4F (82.87 and 76.07 Å, respectively), compared to the *O*‐shaped DIBP3F‐S (74.23 Å). This observed trend, akin to the one observed in the neat films, may result from the reduced miscibility of *S*‐shaped‐OA‐induced PM6 crystallization, aligning with elevated hole mobilities (Figure **5**b). Furthermore, it is observed that EG fluorination‐based blends present a similar yet marginally closer π–π stacking distance compared to V‐DYIC at peaks around *q* = 1.62 Å⁻¹ (*d* = 3.87 Å). Additionally, the former exhibits a considerably larger *CCL* (27.57 Å for V‐DYIC‐4F, 26.81 Å for DIBP3F‐S) than the latter (25.54 Å). Consequently, a dimeric acceptor with an *S*‐shaped conformation contributes to enhanced lamellar order, while the peripheral fluorination strategy improves the order of π‐π stacking. V‐DYIC‐4F amalgamates the benefits of both modification strategies, exhibiting robust crystallinity in both IP and OOP directions. This characteristic proves a systematic strategy in facilitating charge transfer and reducing charge recombination, thereby contributing to the highest FF of the corresponding device.

## 3 Conclusion

In summary, we synthesized two dimeric acceptor materials, namely V‐DYIC and V‐DYIC‐4F, featuring distinct EGs–nonfluorination and difluorination of IC, respectively. The incorporation of vinylene linkage units facilitated the connection of disparate *Y*‐segments in both acceptors. Significantly, both OAs were first confirmed to adopt *S*‐shaped molecular conformations other than *O*‐ or *γ*‐shape, through NMR experiments and theoretical calculations, which is enabled by the strategic introduction of F atoms next to the linking unit. A comparative examination with *O*‐shaped DIBP3F‐S highlights the distinctive coplanar and rigid structure of vinylene‐linked acceptors, deviating from the helical structures associated with *O*‐shaped counterparts. The synergistic effects of conformational attributes and EG fluorination in V‐DYIC‐4F result in augmented absorption spectra, improved π–π stacking, and a meticulously ordered molecular arrangement. This translates to heightened *J*_sc_ and FF in the resultant PSCs. Despite a slightly downshifted LUMO level compared to DIBP3F‐S, V‐DYIC‐4F maintains a noteworthy *V*_oc_. Consequently, PM6:V‐DYIC‐4F‐based devices exhibit superior performance, with a champion efficiency of 18.10% and a minimized energy loss of 0.556 eV, accompanied by enhanced carrier transport efficiency and reduced charge recombination. Moreover, our findings underscore that OAs with *S*‐shaped molecular conformations significantly enhance lamellar order and mitigate nonradiative recombination losses compared to their *O*‐shaped counterparts. Simultaneously, acceptors featuring EG difluorination exhibit improved order in π–π stacking, effectively suppressing energy disorder. Our study demonstrates that it is possible to experimentally determine conformations of OAs and the conformations can have a profound impact on the molecular packing and resulting device performances. These results also provide valuable guidelines for the strategic design of OAs with favorable conformations toward high‐performance PSCs.

## Conflict of Interest

The authors declare no conflict of interest.

## Supporting information

Supporting Information

Supporting Information

## Data Availability

The data that support the findings of this study are available from the corresponding author upon reasonable request.
