# Supplementary material for: Unveiling the Influence of Linkers on Conformations of Oligomeric Acceptors for High‐Performance Polymer Solar Cells
Source: Adv Sci (Weinh). 2024 Aug 29;11(40):2406772. doi: 10.1002/advs.202406772 (PMC11515919; doi:10.1002/advs.202406772)
Supplement: Supplementary file 1 — Supporting Information [file ADVS-11-2406772-s001.pdf]

ADVANCED SCIENCE
Open Access

Supporting Information

for *Adv. Sci.*, DOI 10.1002/advs.202406772

Unveiling the Influence of Linkers on Conformations of Oligomeric Acceptors for High-Performance Polymer Solar Cells

Jingnan Wu, Fengbo Sun, Xunchang Wang, Qiaonan Chen, Leandro R. Franco, Xufan Zheng, C. Moyses Araujo, Renqiang Yang, Donghong Yu* and Ergang Wang**

Supporting Information

Unveiling the Influence of Linkers on Conformations of Oligomeric Acceptors for High-Performance Polymer Solar Cells

Jingnan Wu, Fengbo Sun, Xunchang Wang, Qiaonan Chen, Leandro R. Franco, Xufan Zheng, C. Moyses Araujo, Renqiang Yang, Donghong Yu,* and Ergang Wang**

Materials and Methods.

All the reagents, unless otherwise specified, were purchased from Sigma-Aldrich Co., or Tokyo Chemical Industry Co., Ltd., and were used without further purification. Y-BO-CHO, IC, and IC-FBr were purchased from Acros Pharma Tech Limited. *trans*-1,2-bis(tributylstannyl)ethene was purchased from Tokyo Chemical Industry Co., Ltd.. PM6 was purchased from Solarmer Materials Inc. The general synthetic routes for dimeric acceptors are shown in Scheme S1. BTIC-3FBr was synthesized by using our previously reported method.[1] The detailed synthetic procedures are found in the following session. ^{1}H- and ^{13}C-NMR, spectra were measured in $CDCl_3$ on a Bruker AV 400 MHz FT-NMR spectrometer. ^{19}F NMR and NOESY NMR spectra were measured in $C_2D_2Cl_4$ on a Bruker AV 600 MHz FT-NMR spectrometer. UV-vis spectra of both solution and thin film were recorded on a UV-vis-NIR Spectrophotometer of Agilent Technologies Cary Series. The thin film sample of neat acceptors is obtained with a thickness of around 55 nm by spin-coating from chloroform solution (10 mg mL^{-1}, 1500 rpm for 30s) on quartz substrates. The electrochemical cyclic voltammetry (CV) was taken on a CH-Instruments 650A electrochemical workstation in three electrodes using a glassy carbon as the working electrode, a Pt wire as the counter electrode, and an Ag/Ag^+ reference electrode calibrated using ferrocene/ferrocenium (Fc/Fc^+) redox couple. A 0.1 M tetrabutylammonium hexafluorophosphate (Bu_4NPF_6) in anhydrous acetonitrile solution was the electrolyte, which was bubbled with nitrogen before each measurement.

Synthesis of BTIC-FBr: Y-BO-CHO (400 mg, 0.35 mmol, 1.0 eq) and IC (82 mg, 0.42 mmol, 1.2 eq) were added into a dry 100 mL flask with a magnetic stir bar. After vacuuming and refilling with argon three times, chloroform (40 mL) and pyridine (0.4 mL) were sequentially added to the reaction mixture. They were stirred at room temperature overnight before IC-FBr (102 mg, 0.35 mmol, 1.0 eq.) was added. The resulting mixture was refluxed at 65 ºC for 5 hours. Then the mixture was poured into methanol (300 mL) under stirring for 1-2 h. The precipitate was filtered off and washed with methanol. The resulting crude product was purified by silica gel column chromatography using petroleum ether: dichloromethane (1:1) as eluent to give the pure black solid product (212 mg, 0.12 mmol, Yield: 35%). ^{1}H NMR (400 MHz, Chloroform-*d*) δ 9.06 (s, 2H), 8.61 – 8.58 (m, 1H), 8.29 (d, *J* = 8.2 Hz, 1H), 7.89 (q, *J* = 3.7 Hz, 1H), 7.80 (dd, *J* = 8.2, 6.0 Hz, 1H), 7.70 – 7.66 (m, 2H), 4.72 (q, *J* = 8.2, 7.8 Hz, 4H), 3.13 (dt, *J* = 14.4, 8.0 Hz, 4H), 2.12 (h, *J* = 6.7 Hz, 2H), 1.79 (q, *J* = 7.8 Hz, 4H), 1.42 (h, *J* = 7.6 Hz, 5H), 1.29 (q, *J* = 7.0 Hz, 4H), 1.24 – 0.99 (m, 46H), 0.96 – 0.85 (m, 16H), 0.79 (t, *J* = 6.8 Hz, 8H), 0.65 (t, *J* = 7.3 Hz, 3H), 0.59 (dd, *J* = 10.4, 5.0 Hz, 9H). ^{13}C NMR (151 MHz, Chloroform-

d) δ 188.48, 184.48, 160.78, 158.82, 154.24, 152.95, 147.46, 145.19, 140.64, 140.04, 139.45, 137.97, 137.72, 137.71, 136.91, 136.78, 135.84, 135.27, 135.06, 134.31, 133.55, 131.07, 129.89, 125.21, 123.61, 121.85, 120.99, 119.35, 115.35, 114.95, 113.74, 113.40, 55.76, 39.19, 31.92, 31.57, 31.16, 30.49, 30.37, 29.84, 29.67, 29.63, 29.53, 29.48, 29.35, 28.07, 27.89, 25.47, 25.28, 22.86, 22.69, 22.51, 14.13, 13.98, 13.76.

Synthesis of BTIC-3FBr: see our previous report.[1]

Synthesis of V-DYIC: In a dry 25 flask, 0.03 mmol (1.0 eq) of *trans*-1,2-bis(tributylstannyl)ethene as a linker, 0.072 mmol (2.4 eq) of BTIC-FBr, tris(dibenzylideneacetone)dipalladium(0) ($Pd_2(dba)_3$) (1.10 mg) (4%) and tri(*o*-tolyl)phosphine (P(*o*-Tol)$_3$) (1.10 mg) (12%) were dissolved in anhydrous and degassed toluene (5 mL) under nitrogen atmosphere. The mixture was refluxed with vigorous stirring for 48 h under a nitrogen atmosphere. After cooling down to room temperature, the mixture was poured into 250 mL of methanol, stirred for about 15 minutes, and the precipitate was filtered off and washed with methanol. The resulting crude product was purified by means of silica gel column chromatography using petroleum ether: chloroform: toluene (5:10:1) and petroleum ether: chloroform (1:4) as eluent to give the pure black solid product (48 mg, Yield: 49%). ^{1}H NMR (400 MHz, Chloroform-*d*) δ 9.06 (d, *J* = 33.0 Hz, 2H), 8.63 (d, *J* = 7.8 Hz, 1H), 8.50 (d, *J* = 7.9 Hz, 1H), 7.95 (s, 1H), 7.84 (d, *J* = 7.2 Hz, 1H), 7.67 (t, *J* = 7.5 Hz, 1H), 7.61 (t, *J* = 7.2 Hz, 1H), 7.53 (s, 1H), 4.74 (dp, *J* = 24.4, 13.8, 10.9 Hz, 4H), 3.22 – 3.09 (m, 4H), 2.13 (dt, *J* = 13.5, 7.0 Hz, 2H), 1.81 (dt, *J* = 25.7, 7.7 Hz, 4H), 1.47 (s, 8H), 1.32 – 1.01 (m, 38H), 0.99 – 0.74 (m, 24H), 0.70 – 0.56 (m, 12H). MALDI-TOF-MS m/z:[M] calcd. for $C_{182}H_{210}F_2N_{16}O_4S_{10}$, 3044.39; found: 3044.12.

Synthesis of V-DYIC-4F: A completely comparable procedure as above-described synthesis of V-DYIC, with 0.03 mmol (1.0 eq) of *trans*-1,2-bis(tributylstannyl)ethene as a linker, 0.072 mmol (2.4 eq) of BTIC-3FBr, $Pd_2(dba)_3$ (1.10 mg) (4%) and P(*o*-Tol)$_3$ (1.10 mg) (12%) being dissolved in anhydrous and degassed toluene (5 mL). The product was black solids (45 mg, Yield: 48%). ^{1}H NMR (400 MHz, Chloroform-*d*) δ 9.10 (d, *J* = 29.0 Hz, 2H), 8.55 (d, *J* = 13.5 Hz, 2H), 8.02 (s, 1H), 7.64 (d, *J* = 7.1 Hz, 1H), 7.57 (s, 1H), 4.80 (s, 4H), 3.17 (d, *J* = 47.0 Hz, 4H), 2.19 (s, 2H), 1.97 – 1.74 (m, 4H), 1.53 (d, *J* = 20.4 Hz, 4H), 1.24 (d, *J* = 14.3 Hz, 42H), 1.06 – 0.81 (m, 24H), 0.78 – 0.62 (m, 12H). MALDI-TOF-MS m/z:[M] calcd. for $C_{182}H_{206}F_6N_{16}O_4S_{10}$, 3116.35; found: 3116.14.

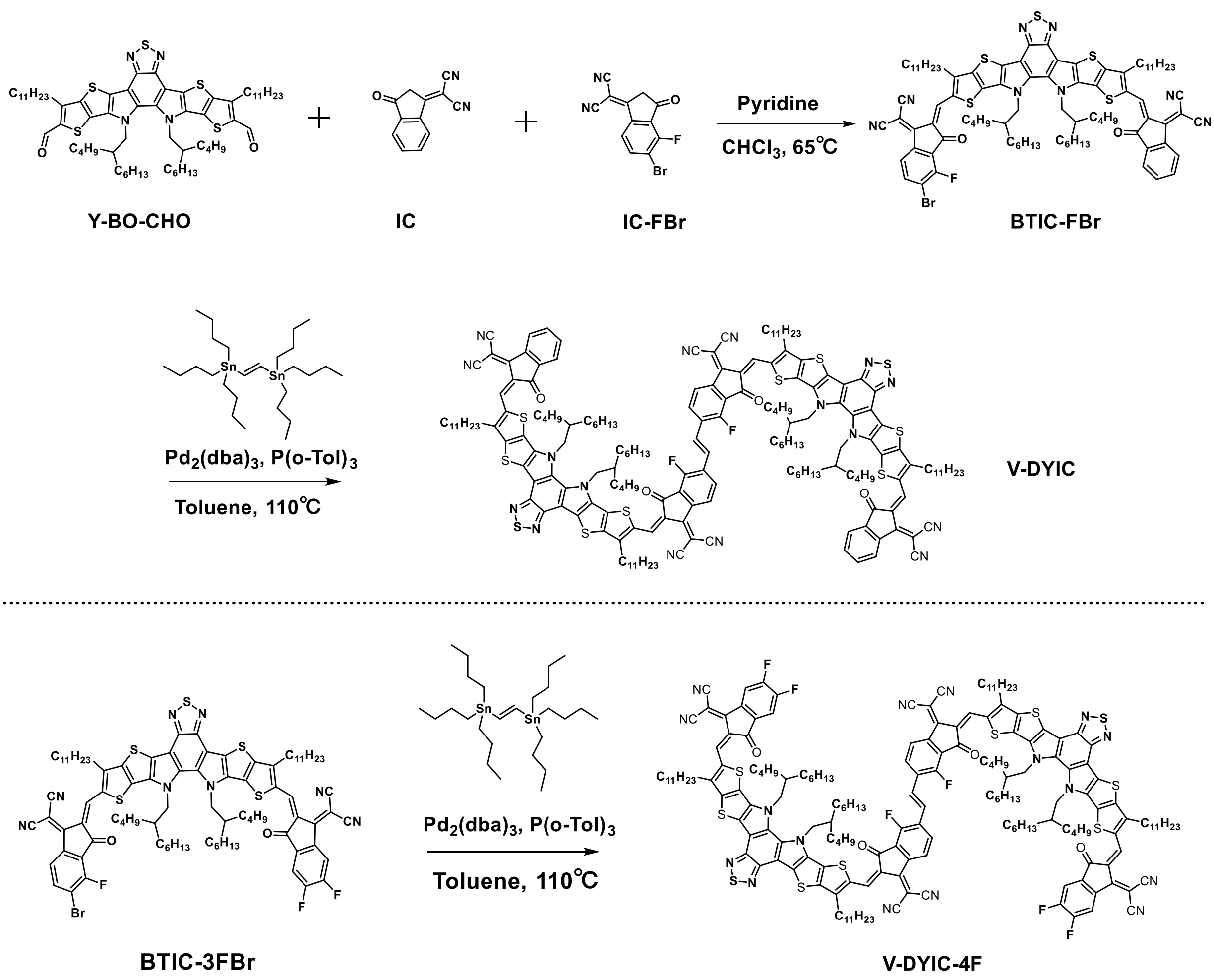

Scheme S1. Synthetic routes of the dimer type acceptors V-DYIC and V-DYIC-4F.

Solar cell device fabrication and characterization. PSC devices were fabricated with a conventional device structure of ITO/PEDOT:PSS/active layer/ Poly[(9,9-bis(3'-((N,N-dimethyl)-N-ethylammonium)-propyl)-2,7-fluorene)-alt-2,7-(9,9-dioctylfluorene)]dibromide (PFN-Br)/Ag. The ITO-coated glass substrates were sonicated successively with detergent, deionized water, acetone, and isopropanol, and then dried with nitrogen flow. Immediately prior to device fabrication, the substrates were cleaned by oxygen plasma for 25 min. Then, the PEDOT:PSS layer was spin-coated onto the ITO and annealed at 150 °C for 15 min. The weight ratio of donor and acceptor remained identical as 1:1.2, while the total concentration of D and A (14 mg/mL) in chloroform and added 1 vol% chloronaphthalene. After solution deposition, the active layer was annealed at 100 °C for 5 min. Then PFN-Br as an electron transport layer material were dissolved in methanol to a concentration of 0.5 mg/mL and were spin-coated on top of the active layer (spinning rate: 2000 rpm for 30s). Finally, Ag (100 nm) was evaporated onto the active layer at a vacuum of ~2×10^{-4} Pa to form the top electrode. The effective area of

the device is 0.042 cm^2. The current-voltage (*J-V*) characteristics were measured with a Keithley 2420 source measurement unit. The PSCs were measured under an irradiation intensity of 100 mW/cm2 (AM 1.5 G) by a Newport solar simulator. The EQE spectra were analyzed using a certified Newport IPCE measurement system. The highly sensitive EQE was measured by using an integrated system (PECT-600, Enlitech), where the photocurrent was amplified and modulated by a lock-in instrument. Electroluminescence (EL) quantum efficiency (EQE_{EL}) measurements were performed by applying external voltage/current sources through the devices (ELCT-3010, Enlitech).

Space-charge-limited current (SCLC) measurement: The carrier mobilities of the PM6:OA were determined by fitting the dark current to the SCLC model. The device structures were glass/ITO/PEDOT:PSS/BHJ/MoO_3/Ag (Hole-only devices) and glass/ITO/ZnO/BHJ/PDINN/Ag (Electron-only devices). The electric-field dependent SCLC mobility was estimated using Equation:

$$J(V) = \frac{9}{8}\varepsilon_0\varepsilon_r\mu_0 \exp(0.89\beta\sqrt{\frac{V-V_{bi}}{L}})\frac{(V-V_{bi})^2}{L^3}$$

where μ_0 is zero-field mobility, L is the film thickness, J is the dark current density, V is the voltage, ε_0 is vacuum permittivity, ε_r is the dielectric constant, and β is the field activation factor.

Contact Angle Measurement. Contact angles were measured with a contact angle meter (GBX DIGIDROP). The solution of each organic material was spin-coated on cleaned ITO substrates. Droplets of water and dimethoxyethane were dripped onto the different films. According to the Owens-Wendt method, surface tension could be divided into dispersive and polar components:

$\gamma = \gamma^d + \gamma^p$

Furthermore, the dispersive and polar surface tension were calculated through the formula below based on the contact angles obtained by two solvents. $\gamma_L(1 + \cos\theta) = 2(\gamma_S^d\gamma_L^d)^{1/2} + 2(\gamma_S^p\gamma_L^p)^{1/2}$, where θ is the contact angle of a specific solvent, γ_L is the surface tension of the solvent, γ_S^d and γ_S^p refer to the dispersive and polar surface tension of the solid, respectively, and γ_L^d and γ_L^p refer to the dispersive and polar surface tension of the solvent, respectively. Thus, the unknown values $\gamma S\ d$ and $\gamma L\ p$ can be solved by combining two equations obtained by contact angle measurement of two different solvents.

Grazing Incidence Wide Angle X-Ray Scattering (GIWAXS): 2D-GIWAXS experiments were carried out on a GANESHA 300XL+ system from JJ X-ray. The instrument is equipped with a Pilatus 300K detector, with pixel size of 172 μm × 172 μm. The X-ray source is a Genix 3D Microfocus Sealed Tube X-Ray Cu-source with integrated Monochromator (30 W). The wavelength used is λ = 1.5418 Å. The detector moves in a vacuum chamber with sample-to-detector distance varied between 0.115 m and 1.47 m depending on the configuration used, as calibrated using silver behenate (d001 = 58.380 Å). The minimized background scattering plus high-performance detector allows for a detectable q-range varying from 3×10^{-3} to 3 $Å^{-1}$ (0.2 to 210 nm). The sample was placed vertically on the goniometer and tilted to a glancing angle of 0.2° with respect to the incoming beam. A small beam was used to get a better resolution. The accumulation time was 30 minutes for each measurement. In-plane and out-of-plane line cuts were obtained using the SAXSGUI program.

Theoretical Modelling: We investigated the electronic structure of the oligomers through Density Functional Theory (DFT) calculations. Molecular geometries were optimized using the B3LYP-GD3BJ/6-311+G(d,p) [2-4] theory level to attain minimum energy configurations. The energy levels (HOMO and LUMO) of the systems were determined by considering the total Gibbs Free Energy of neutral and charged species within the diabatic approximation, incorporating the structural reorganization energy of charged species. Additionally, nuclear magnetic shieldings were computed using the Gauge Including Atomic Orbitals (GIAO)[5] approximation. Chemical shifts (δ) of proton were referenced to tetramethylsilane (TMS), with its δ value (31.6 ppm) calculated at the same theory level and then subtracted from the molecule values to derive the chemical shifts. Throughout the calculations, environmental effects were considered via the dielectric constant of chloroform (ε=4.71) using the implicit SMD[6] model. For computational efficiency, methyl groups replaced alkyl side chains. All calculations were performed using the Gaussian 16 program (Rev C.01).[7]

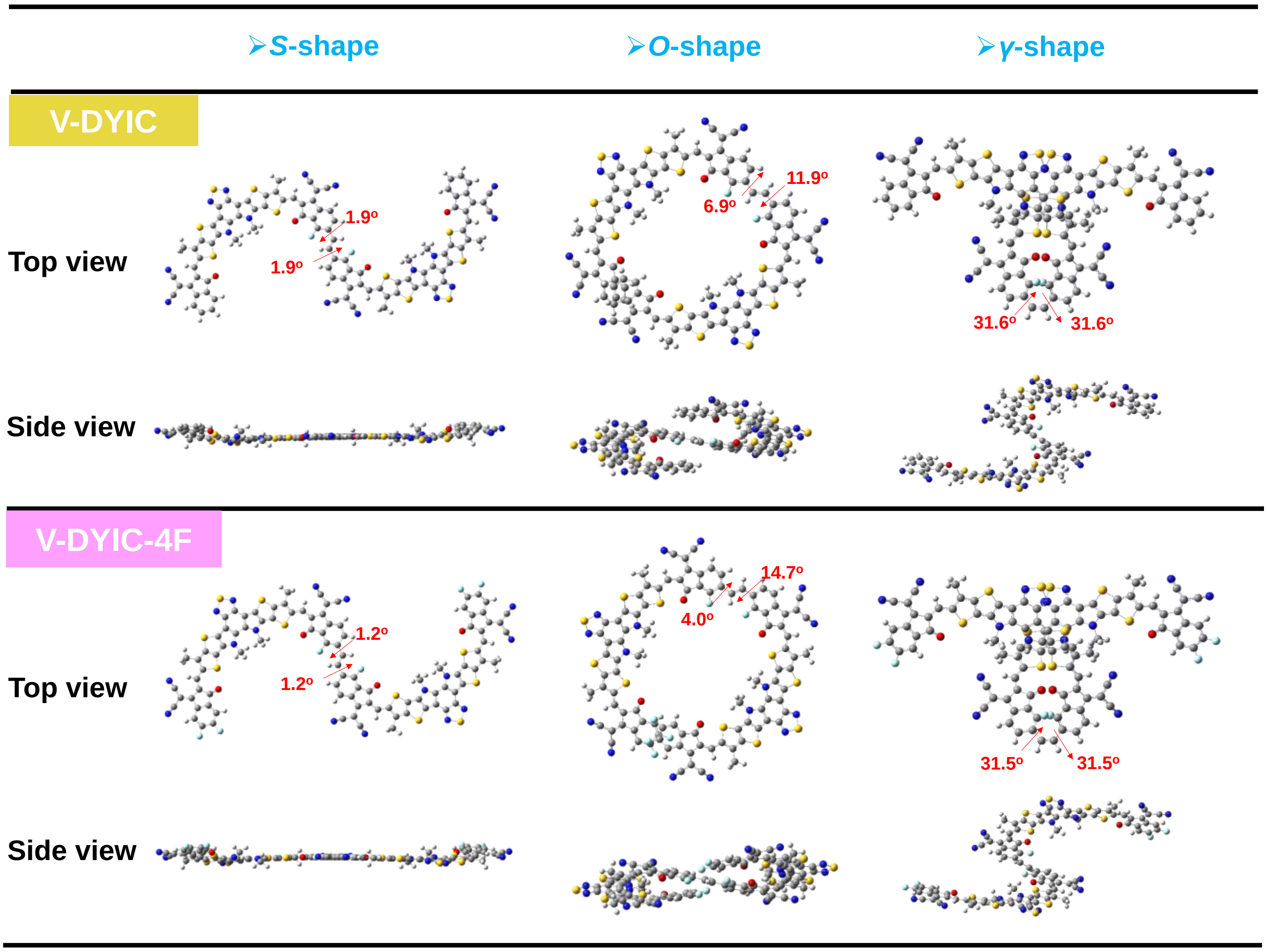

Figure S1. DFT-simulated molecular geometry from the top and side-view of the molecular models for V-DYIC and V-DYIC-4F.

Table S1. Theoretical electronic energy of V-DYIC and V-DYIC-4F under different conformations.

	Conformers	Electronic Energy (E_0) (kcal/mol)
V-DYIC	*S*	0.00
	O	0.18
	γ	5.59
	Reference for E_0	-5722578.27
V-DYIC-4F	*S*	0.00
	O	0.79
	γ	5.71
	Reference for E_0	-5971728.43

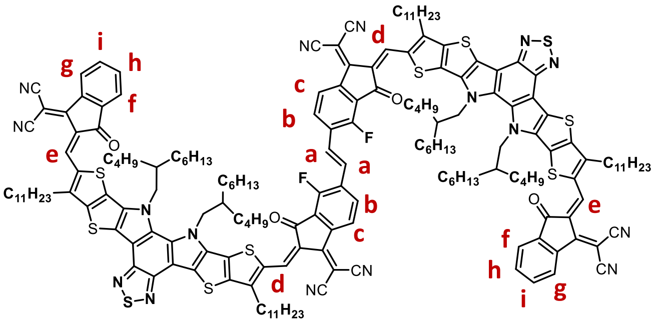

V-DYIC

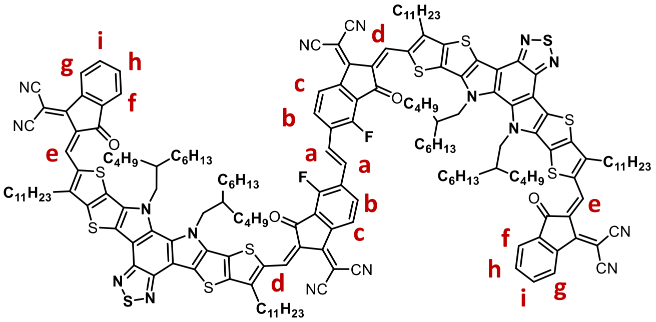

V-DYIC-4F

Figure S2. Labelled protons of V-DYIC and V-DYIC-4F.

Table S2. Theoretical and experimental chemical shifts (in ppm) of H protons of V-DYIC with different conformations in the $CDCl_3$ solution.

Proton[a]	DFT		EXP.	Δ(DFT – EXP.)	
	O-shape	*S*-shape		*O*-shape	*S*-shape
a	8.55	8.06	7.53	1.02	0.53
a	7.50	8.06	7.53	-0.03	0.53
b	7.81	7.82	7.95	-0.14	-0.13
c	8.54	8.57	8.51	0.03	0.06
d	8.99	8.94	9.09	-0.10	-0.15
e	9.03	8.93	9.04	-0.01	-0.11
g	7.99	8.01	7.85	0.14	0.16
f	8.74	8.82	8.63	0.11	0.19
h	7.58	7.82	7.67	-0.09	0.15
i	7.54	7.78	7.61	-0.07	0.17

[a]Refer to the structures in Figure S2.

Table S3. Theoretical and experimental chemical shifts (in ppm) of H protons of V-DYIC-4F with different conformations in the $CDCl_3$ solution.

Proton[a]	DFT		EXP.	Δ(DFT – EXP.)	
	O-shape	*S*-shape		*O*-shape	*S*-shape
a	8.46	8.07	7.57	0.89	0.50
a	7.56	8.07	7.57	-0.01	0.50
b	7.82	7.83	8.02	-0.20	-0.19
c	8.58	8.57	8.54	0.04	0.03
d	9.03	8.95	9.14	-0.11	-0.19
e	8.97	8.88	9.06	-0.09	-0.18
f	7.85	7.77	7.65	0.20	0.12
g	8.74	8.63	8.56	0.18	0.07

[a]Refer to the structures in Figure S2.

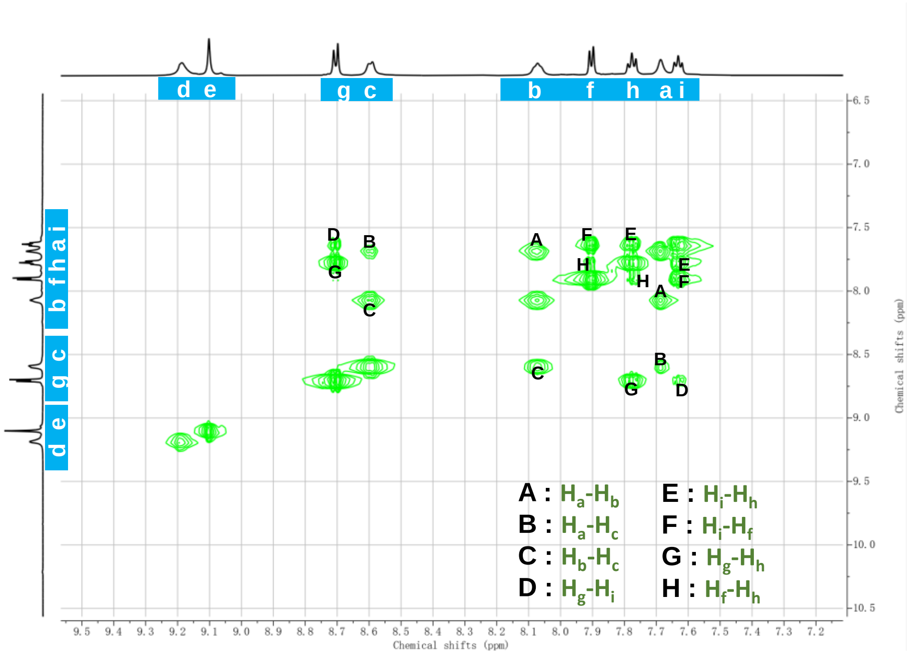

Figure S3. ^{1}H-^{1}H NMR spectrum (600 MHz) of V-DYIC in the $C_2D_2Cl_4$ solution at 313 K.

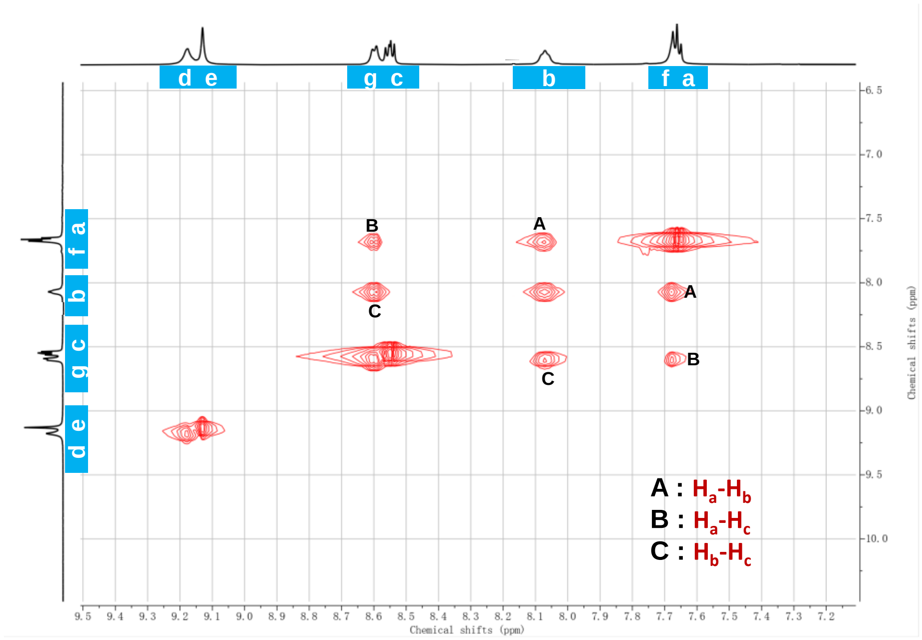

Figure S4. ^{1}H-^{1}H NMR spectrum (600 MHz) of V-DYIC-4F in the $C_2D_2Cl_4$ solution at 313 K.

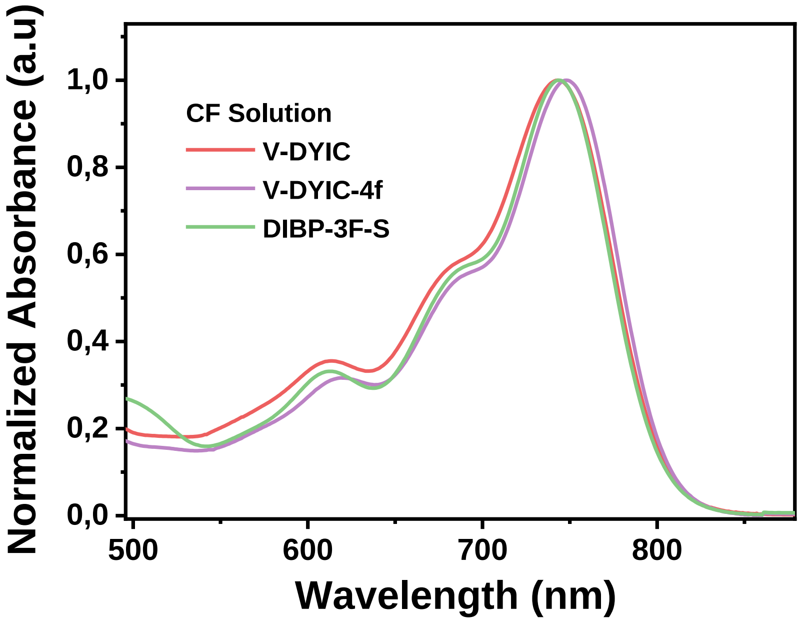

Figure S5. Normalized UV-vis absorption spectra of V-DYIC, V-DYIC-4F and DIBP3F-S in chloroform (CF) solution

Table S4. Basic optical properties of V-DYIC, V-DYIC-4F and DIBP3F-S.

Acceptor	$\lambda_{max}^{sol.}$ (nm)	λ_{max}^{film} (nm)	λ_{onset}^{sol} (nm)	λ_{onset}^{film} (nm)	E_g^{opt} (eV) [a]	ε (10^5 cm^{-1})
V-DYIC	742	803	802	891	1.39	1.94
V-DYIC-4F	749	817	807	904	1.37	1.99
DIBP3F-S	743	804	804	899	1.38	2.01

[a]Calculated as $1240/\lambda_{onset}^{film}$.

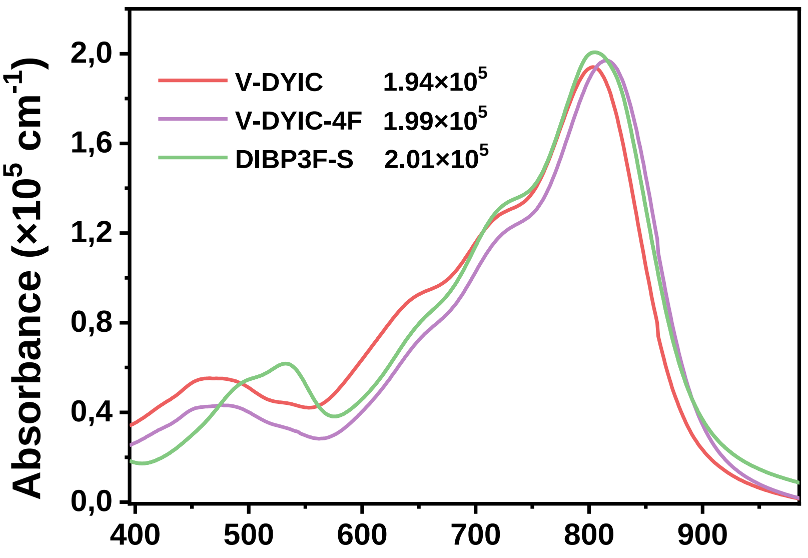

Figure S6. The absorption coefficients of neat films (50 nm) of V-DYIC, V-DYIC-4F, and DIBP3F-S.

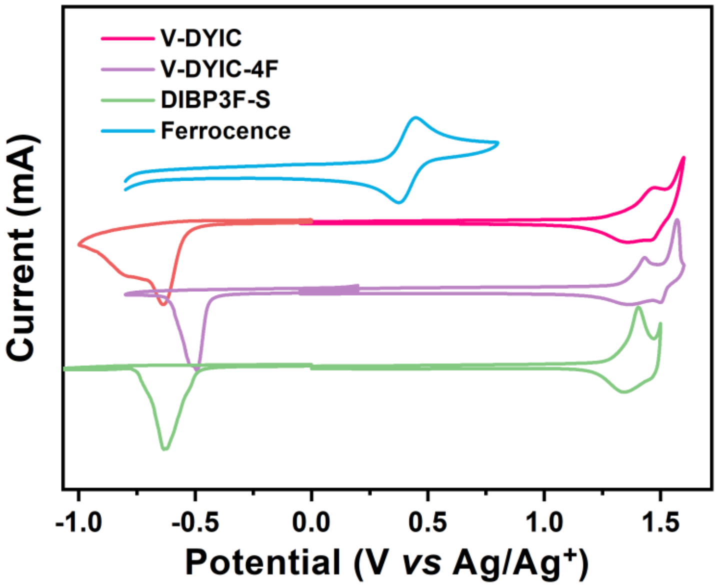

Figure S7. Cyclic voltammograms of the OAs in films on a glassy carbon electrode measured in a 0.1 M Bu_4NPF_6 acetonitrile solution at a scan rate of 50 mV/s.

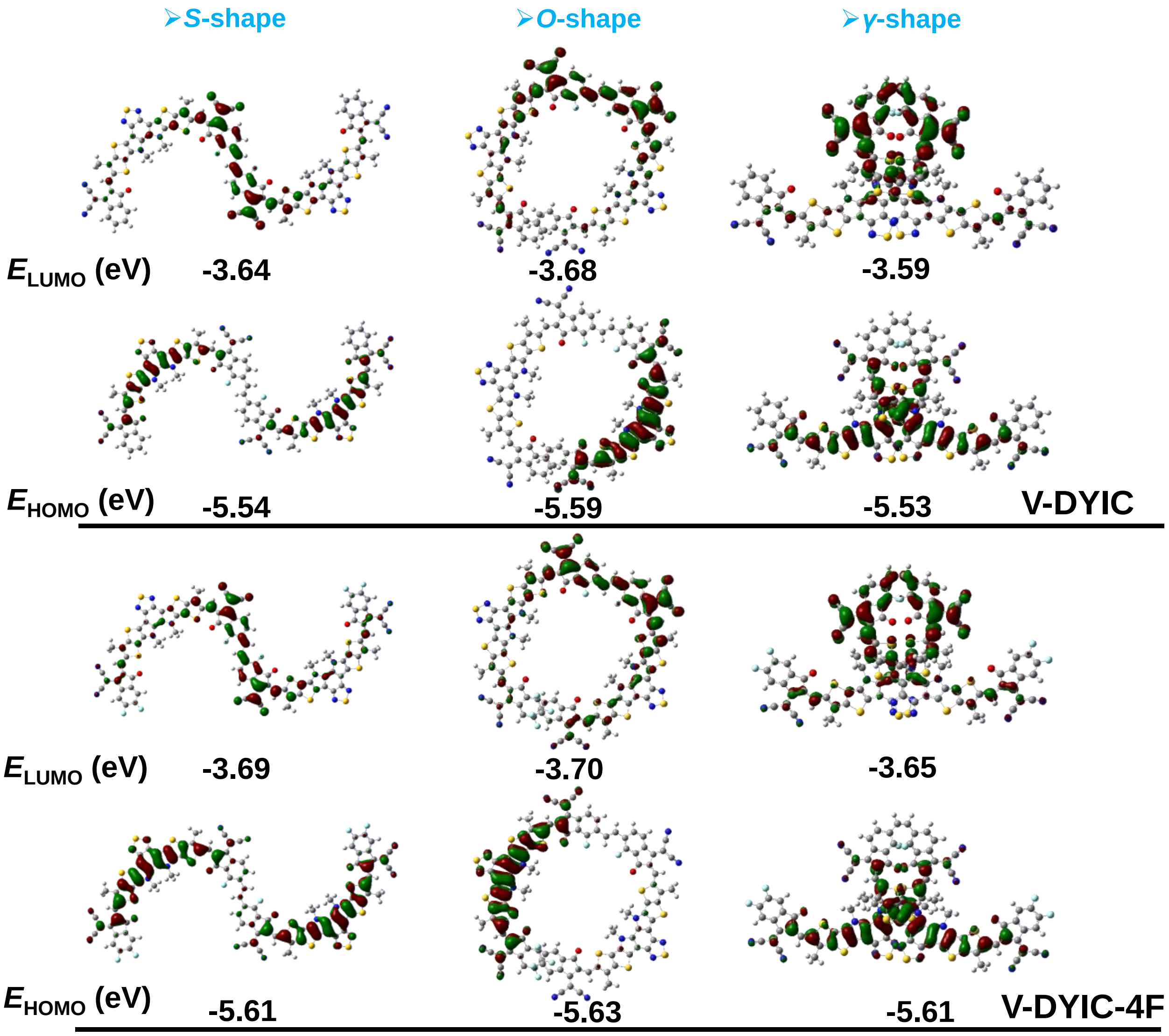

Figure S8. DFT-simulated electronic density distributions of the frontier molecular orbitals for the represented *S*-, *O*- and γ-shape conformation of V-DYIC and V-DYIC-4F molecular models, calculated by B3LYP-D3BJ/6-311G(d, p). Basic set with methyl groups in replacing alkyl substituents to reduce the loads of calculations.

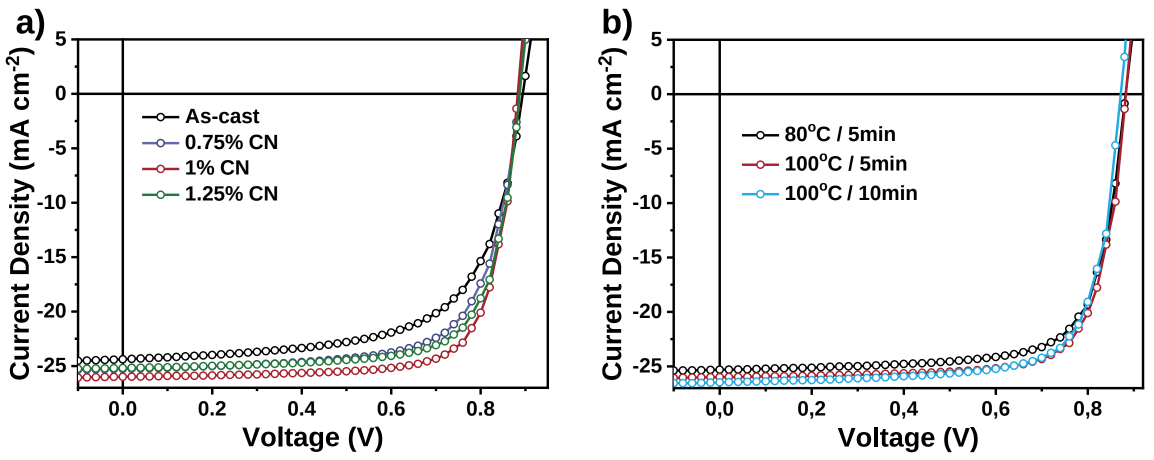

Figure S9. *J-V* characteristics of the PSCs based on PM6:V-DYIC-4F with different contents of CN and thermal annealing treatments under the irradiation of AM 1.5G, 100 mW/cm^2.

Table S5. Photovoltaic performance parameters of the PSCs based on PM6:V-DYIC-4F with different contents of DIO by annealing at 100 °C for 5 min under the irradiation of AM 1.5G, 100 mW/cm^2.

CN	V_{oc} [V]	J_{sc} [mA/cm^2]	FF [%]	PCE [%]
W/O	0.895	24.39	64.9	14.16
0.75% CN	0.887	25.27	70.6	15.82
1% CN	0.883	25.99	75.7	17.28
1.25% CN	0.887	25.16	73.3	16.35

Table S6. Photovoltaic performance parameters of the PSCs based on PM6:V-DYIC-4F with different annealing temperature and time, under the irradiation of AM 1.5G, 100 mW/cm^2.

Temperature/time	V_{oc} [V]	J_{sc} [mA/cm^2]	FF [%]	PCE [%]
85°C/5min	0.882	25.31	74.3	16.59
100°C/5min	0.883	26.35	77.8	18.10
100°C/10min	0.872	26.47	74.8	17.27

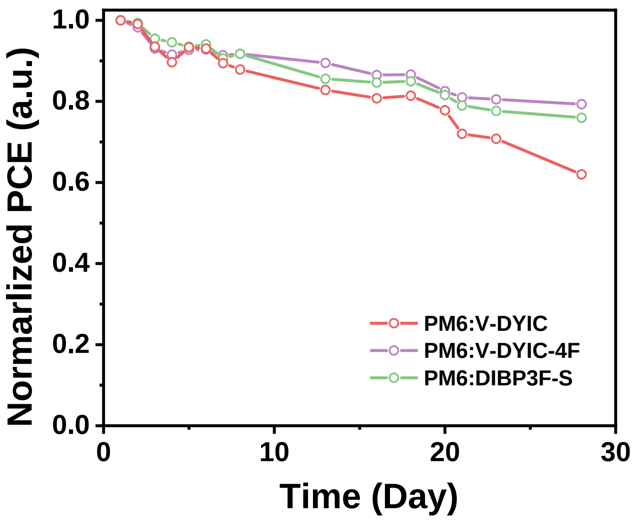

Figure S10. Normalized PCEs of the PM6:OA-based PSCs after the aged at 65°C.

Table S7. The energy loss of PM6:OA-based PSCs.

PM6:OA	E_g (eV)	qV_{oc}^{SQ} (eV)	qV_{oc}^{rad} (eV)	ΔE_1 (eV)	ΔE_2 (eV)	ΔE_3 (eV)	E_{loss} (eV)	EQE_{EL} /10^{-4}
V-DYIC	1.463	1.194	1.121	0.268	0.074	0.216	0.558	2.37
V-DYIC-4F	1.439	1.172	1.105	0.267	0.067	0.222	0.556	1.88
DIBP3F-S	1.454	1.187	1.117	0.269	0.067	0.236	0.573	1.07

$\Delta E_1 = E_g - qV_{oc}^{SQ}$; $\Delta E_2 = q\Delta V_{oc}^{rad,\ below\ gap}$; $\Delta E_3 = q\Delta V_{oc}^{non\text{-}rad}$ [8].

Table S8. Charge mobilities of the device's active layers were measured by space-charge-limited current method.

PM6:OA	μ_h [cm^2 V^{-1} s^{-1}]	μ_e [cm^2 V^{-1}s^{-1}]	μ_e/μ_h
V-DYIC	-	1.74×10^{-4}	-
V-DYIC-4F	-	2.88×10^{-4}	-
DIBP3F-S	-	3.82×10^{-4}	-
PM6:V-DYIC	3.33×10^{-4}	5.40×10^{-4}	1.92
PM6:V-DYIC-4F	5.35×10^{-4}	8.46×10^{-4}	1.58
PM6:DIBP3F-S	2.78×10^{-4}	7.06×10^{-4}	2.54

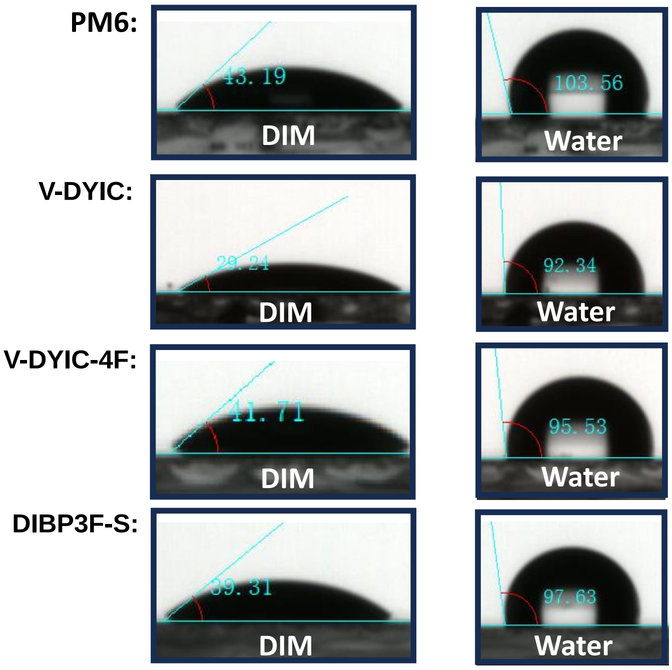

Figure S11. The contact angle images of PM6, V-DYIC, V-DYIC-4F and DIBP3F-S films performed by using deionized water (H_2O) and dimethoxyethane (DIM) as wetting liquids.

Table S9. Contact angles and surface energy on various neat films.

Solid surface	H_2O contact angle (°)	DIM contact angle (°)	Surface energy [mN/m][a]	$(\gamma_D^{-2}-\gamma_A^{-2})^2$
PM6	103.56	43.19	40.47	/
V-DYIC	92.34	29.24	45.18	0.1296
V-DYIC-4F	95.53	41.71	39.16	0.0107
DIBP3F-S	97.63	39.31	41.22	0.0034

[a]Surface energy was converted from contact angle values based on the Owens, Wendt, Rabel, and Kaelble (OWRK) equation.

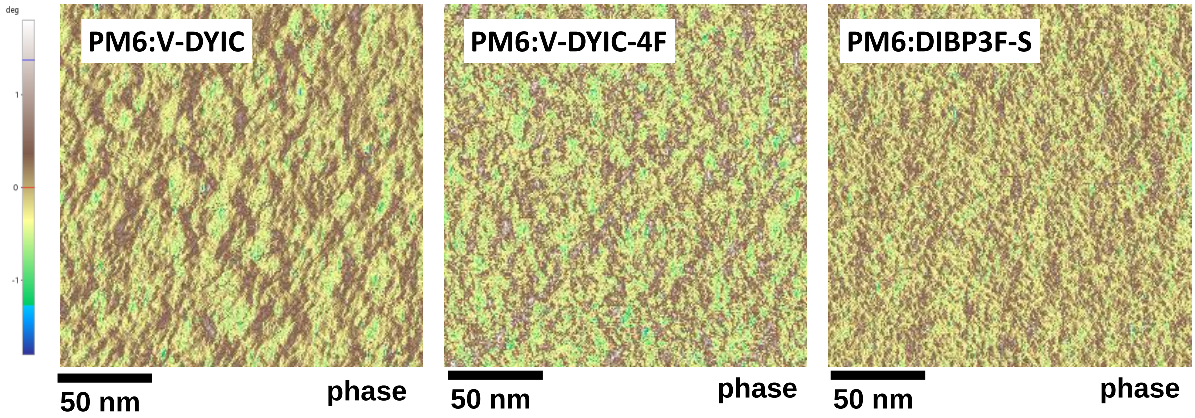

Figure S12. Tapping mode AFM topography phase-image of blend films of PM6:V-DYIC, PM6:V-DYIC-4F and PM6:DIBP3F-S.

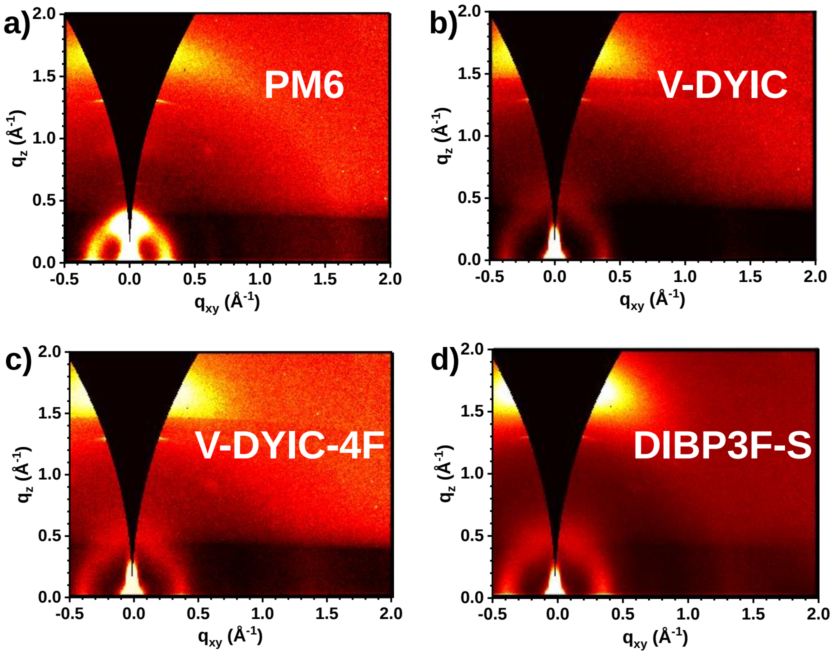

Figure S13. 2D GIWAXS profiles of neat films of PM6, V-DYIC, V-DYIC-4F, and DIBP3F-S, respectively.

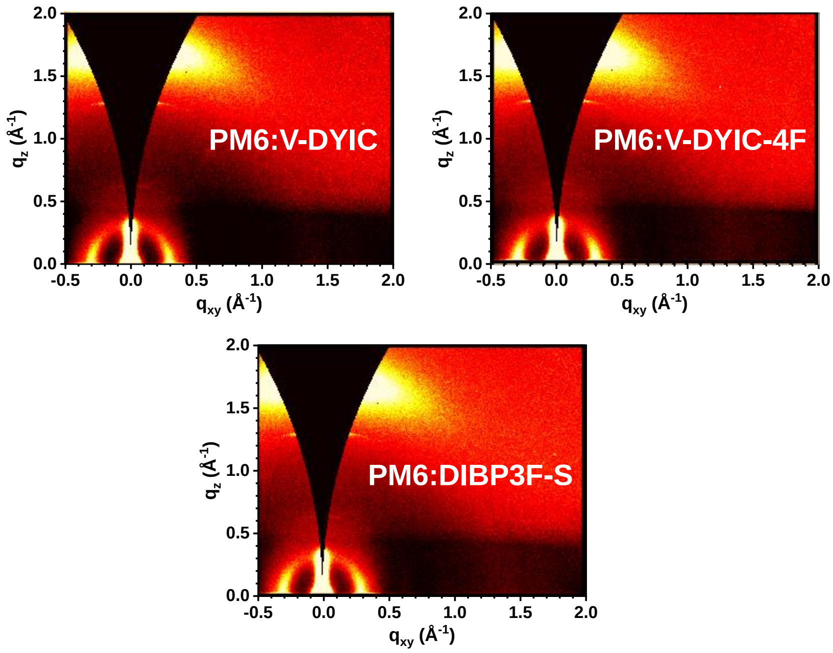

Figure S14. 2D GIWAXS profiles of blend films of PM6:V-DYIC, PM6:V-DYIC-4F and PM6:DIBP3F-S.

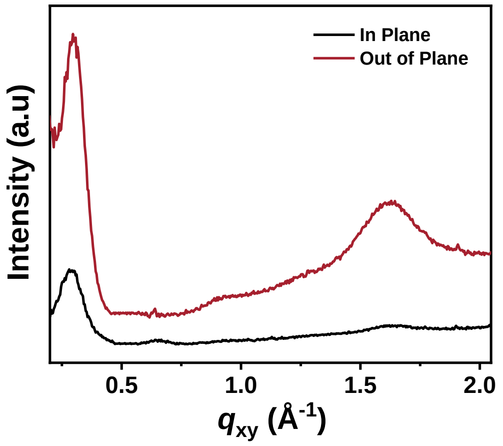

Figure S15. 1D line cuts of PM6 neat films form 2D GIWAXS patterns.

Table S10. Summary of the crystal coherence length (*CCL*) and *d*-spacing of neat OAs and corresponding PM6-based blends.

Film		**LOCATION ($Å^{-1}$)**	**FWHM ($Å^{-1}$)**	**CCL (Å)**	***d*-spacing (Å)**
V-DYIC	OOP (010)	1.61	0.28	20.33	3.89
V-DYIC-4F		1.63	0.27	21.21	3.84
DIBP3F-S		1.61	0.26	21.35	3.91
PM6		1.63	0.21	26.70	3.86
PM6:V-DYIC		1.62	0.22	25.54	3.88
PM6:V-DYIC-4F		1.62	0.21	27.57	3.87
PM6:DIBP3F-S		1.63	0.21	26.81	3.86
V-DYIC	IP (100)	0.38	0.10	56.54	16.57
V-DYIC-4F		0.38	0.09	61.65	16.39
DIBP3F-S		0.37	0.13	42.77	16.80
PM6		0.29	0.10	55.80	21.97
PM6:V-DYIC		0.31	0.07	82.87	20.53
PM6:V-DYIC-4F		0.31	0.07	76.07	20.43
PM6:DIBP3F-S		0.31	0.08	74.23	20.40

References

[1] J. Wu, Z. Ling, L. R. Franco, S. Y. Jeong, Z. Genene, J. Mena, S. Chen, C. Chen, C. M. Araujo, C. F. N. Marchiori, J. Kimpel, X. Chang, F. H. Isikgor, Q. Chen, H. Faber, Y. Han, F. Laquai, M. Zhang, H. Y. Woo, D. Yu, T. D. Anthopoulos and E. Wang, *Angew. Chem. Int. Ed.*, **2023**, 62, e202302888.

[2] A. D. Becke, *J. Chem. Phys.*, **1993,** 98, 5648.

[3] S. Grimme, S. Ehrlich and L. Goerigk, *J. Comp. Chem.*, **2011**, 32, 1456.

[4] K. Raghavachari, J. S. Binkley, R. Seeger, and J. A. Pople, *J. Chem. Phys.*, **1980**, 72, 650.

[5] D) K. Wolinski, , F. H. James, and P. Peter, *J. Am. Chem. Soc.* **1990**, 112, 8251.

[6] A. V. Marenich, J. C. Christopher, and G. T. Donald, *J. Phys. Chem. B*, **2009**, 113, 6378.

[7] M. J. Frisch, G. W. Trucks, H. B. Schlegel, G. E. Scuseria, M. A. Robb, J. R. Cheeseman, G. Scalmani, Gaussian 16, revision C. 01. **2016**.

[8] S. Liu, J. Yuan, W. Deng, M. Luo, Y. Xie, Q. Liang, Y. Zou, Z. He, H. Wu and Y. Cao, *Nat. Photon.*, **2020**, 14, 300.
